# High-Density Lipoproteins and Mediterranean Diet: A Systematic Review

**DOI:** 10.3390/nu13030955

**Published:** 2021-03-16

**Authors:** Elena Grao-Cruces, Lourdes M. Varela, Maria E. Martin, Beatriz Bermudez, Sergio Montserrat-de la Paz

**Affiliations:** 1 Department of Medical Biochemistry, Molecular Biology and Immunology, School of Medicine, University of Seville, 41009 Seville, Spain; egrao@us.es (E.G.-C.); delapaz@us.es (S.M.-d.l.P.); 2 Institute of Biomedicine of Seville, IBiS, Universitary Hospital Virgen del Rocio/CSIC/University of Seville, 41013 Seville, Spain; 3 Department of Medical Physiology and Biophysics, University of Seville, 41009 Seville, Spain; 4 Department of Cell Biology, Faculty of Biology, University of Seville, 41012 Seville, Spain; mariamartin@us.es (M.E.M.); bbermudez@us.es (B.B.)

**Keywords:** high-density lipoprotein, lipidome, proteome, Mediterranean diet, olive oil

## Abstract

Cardiovascular disease (CVD) is the leading cause of global mortality and the study of high-density lipoproteins (HDL) particle composition and functionality has become a matter of high interest, particularly in light to the disappointing clinical data for HDL-cholesterol (HDL-C) raising therapies in CVD secondary prevention and the lack of association between HDL-C and the risk of CVD. Recent evidences suggest that HDL composition and functionality could be modulated by diet. The purpose of this systematic review was to investigate the effect of Mediterranean diet (MD) on changes in HDL structure and functionality in humans. A comprehensive search was conducted in four databases (PubMed, Scopus, Cochrane library and Web of Science) and 13 records were chosen. MD showed favorable effects on HDL functionality, particularly by improving HDL cholesterol efflux capacity and decreasing HDL oxidation. In addition, HDL composition and size were influenced by MD. Thus, MD is a protective factor against CVD associated with the improvement of HDL quality and the prevention of HDL dysfunctionality.

## 1. Introduction

Cardiovascular disease (CVD) remains the leading cause of morbidity and mortality worldwide. It is well known that high concentrations of circulating high-density lipoprotein-cholesterol (HDL-C) are inversely correlated with the risk of CVD [1]. While raising HDL-C is a theoretically attractive target, there is no evidence from randomized trials that increasing plasma HDL-C concentrations reduces CVD risk [2].

Several studies have shown that high-density lipoproteins (HDLs) are highly heterogeneous in size, shape, density, lipid and protein composition [3]. HDL particles undergo continuous remodeling through interactions with other circulating lipoproteins and tissues [4]. HDL-associated proteins have been considered until now to predominate in determining the particle structure and biological functions: cholesterol removal, anti-inflammatory, antioxidant and endothelial cell protection [5]. For example, paraoxonase 1 (PON1) is a HDL-associated protein mediating HDL functionality that has been negatively correlated with unfavorable outcome in stroke patients [6]. However, the latest cutting edge lipidomic technology has revealed important roles of lipid components in HDL function. Sphingomielin-1-phosphate (S1P) is the most studied bioactive lipid bound to HDL and it has been negatively correlated with the severity of coronary artery disease [7]. Recent evidence linked increased odds of acute coronary syndrome to low cholesterol efflux capacity (CEC), pro-oxidant and pro-inflammatory HDL particles and low HDL levels of S1P [8]. In addition, HDL-C was found to be highly related to cardiovascular risk when it was carried by small HDLs, suggesting that cardiovascular risk is highly influenced by HDL size [9]. Taken all together, it is becoming clear that circulating HDL-C plasma concentrations is not an appropriate marker of CVD risk and therefore do not represent a reliable therapeutic target. Targeting HDL functionality rather than HDL-C concentrations may represent a more promising therapeutic target.

Different strategies are followed in order to modify plasma HDL-C and cardiovascular health. Healthy diets have beneficial effects on lipid profile, replacing saturated fatty acids (SFA) with either monounsaturated (MUFA) or polyunsaturated fatty acids (PUFA) have been shown to reduce plasma HDL-C [10]. This objective is achieved with Mediterranean diet (MD), rich in olive oil (OO) fruits and vegetables, nuts, legumes and whole cereals, fish and red wine. Data from observational and randomized controlled trials supports that MD protects against CVD, MD has showed capacity to improve lipid profile related to HDL-C [11]. The purpose of this systematic review was to investigate the effect of the MD on changes in HDL structure and functionality in humans.

## 2. Materials and Methods

This systematic review was conducted following the Preferred Reporting Items for Systematic reviews and Meta-analysis (PRISMA) [12]. This review was registered on PROSPERO (Registration No. CRD42020218784). Available from: https://www.crd.york.ac.uk/prospero/display_record.php?ID=CRD42020218784 (accessed on 23 February 2021).

### 2.1. Searching Strategy

A comprehensive search was conducted in four databases (PubMed, Scopus, Web of Science and Cochrane Library), searching all years of record up until October 2020. The language restriction was English and Spanish. Search terms were categorized into 3 key concepts: study population, HDL modulation, and nutritional intervention; specific terms used were: (adult OR “middle-aged” OR young OR m?n OR wom?n OR obese OR healthy) AND (“Mediterranean diet” OR “olive oil”) AND (“HDL remodel*” or “HDL function*” OR “HDL dysfunction*” OR “HDL change” OR “HDL component”), respectively. Whereas, reference lists of retrieved articles were manually searched for relevant publications (Table 1).

### 2.2. Selection Criteria

Published studies included in this review were require to adhere to the following criteria: (1) original research; (2) adult human studies; (3) articles including HDL changes in composition, HDL component modifications or HDL functionality changes; (4) dietary intervention with MD or MD-related foods; (5) obese, dyslipidemic and healthy humans.

### 2.3. Data Extraction and Reliability

The PRISMA recommendations were followed. Firstly, titles were screened and abstracts were analyzed in order to identify relevant articles. Then, articles chosen were downloaded and reviewed in detail by different researchers.

## 3. Results

### 3.1. Search and Selection of Studies

**Figure 1:**
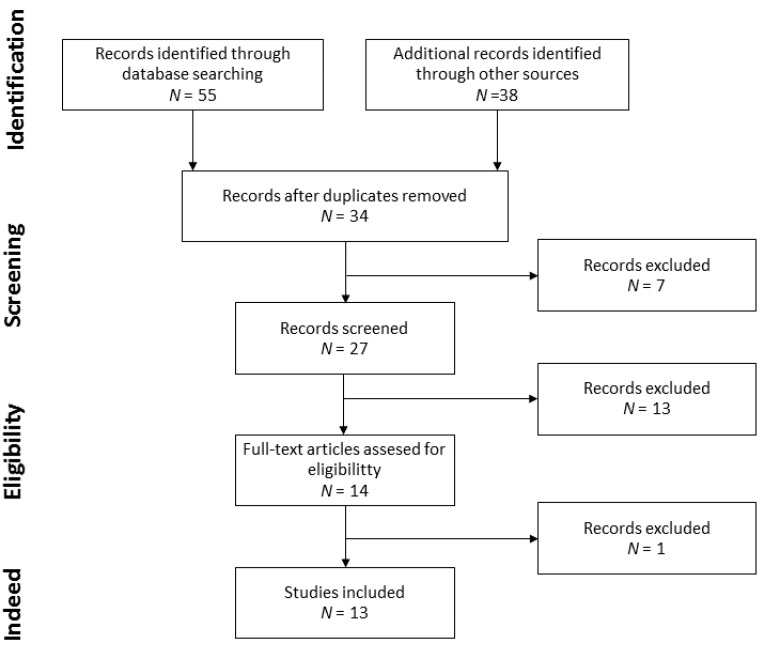
Flow diagram of record selection.

The searching and selection strategies are detailed in Figure 1. A total of 55 records were identified through database searching and 38 through other sources, search of reference list of retrieved articles. Duplicates were removed and a set of 34 records were left to select the ones to be screened. From these 34, 7 records were excluded due to article type and 27 records were susceptible of full-text assessment. Consequently, 13 records were excluded with criteria, and 14 records were assessed for eligibility. Finally, 1 record was excluded because of the methodology used and 13 records were analyzed.

### 3.2. Comparison between Studies

Nine different study populations have been found in the records chosen, whereas, ten nutritional interventions were studied (Table 2). Two records studied the same 296 individuals with three nutritional interventions [13,14]. Otherwise, after the interventions, Hernaez et al., 2019, made an evaluation of foods consumed by 196 individuals from the initial study population, in order to create different groups [13]. Individuals in each study population were different between studies, healthy individuals were included in a total of 5 study populations [15,16,17,18,19], and the rest of the study populations included high cardiovascular risk individuals, which suffered from dyslipidemia, obesity or hypercholesterolemia [13,14,20,21,22,23,24,25].

Intervention time varied between studies. The longest intervention time was 1 year [13,14] and the shortest 4 days [17]. 

### 3.3. HDL Functionality Modulation

A set of 7 records measured cholesterol efflux capacity (CEC), CEC measurements were conducted on THP-1 monocyte-derived macrophages [13,14,15,18,20], on J-774A.1 macrophages [22,23,24,25] and on primary cultures [19]. PON, lecithin cholesterol acyl transferase (LCAT), and cholesteryl ester transfer protein (CETP) activities were determined in a set of 5 [13,14,16,20,22,23,24,25], 3 [15,16,22,23,24,25] and 4 [13,14,15,16] populations, respectively, by enzymatic activity measurements.

Specific MD-related foods were able to modulate HDL functions (Table 3). An increase of CEC was showed after virgin OO (VOO), whole grains and nuts [13] eicosapentaenoic acid (EPA) [20] and extra VOO [18] consumption compared to baseline. Enzyme activity was variable after different interventions: CETP activity was decreased by diets enriched with legumes, fish and VOO [13]. PON1 activity was increased by nut, legume and fish enriched diets [13], EPA consumption [20], lycopene rich and supplemented diets [16] and functional VOO with thyme (FVOOT) intervention [22,23,24,25]. LCAT activity increased with lycopene supplemented diets [16]. Although there was no change after VOO, functional VOO (FVOO and FVOOT) interventions in LCAT activity, it was significantly higher after FVOOT in comparison with VOO intervention [22,23,24,25].

### 3.4. HDL Oxidation

HDLs are affected by oxidative modifications and the fatty acids contained in HDL-associated lipids are the most susceptible components to oxidation. HDL oxidation state (Table 4) is related to HDL functionality. HDL oxidation rate was measured as equivalents of malondialdehyde production [14,19], and other HDL oxidation-related parameters were measured by Hernaez et al. 2017: HDL capacity to prevent low-density lipoprotein (LDL) oxidation and HDL resistance to oxidation in a pro-oxidant environment [14]. After a VOO-enriched traditional MD, HDL antioxidant properties were improved, lower HDL oxidation rate, higher HDL resistance to oxidation and higher prevention of LDL oxidation were found [14]. In addition, linoleic supplemented diet showed capacity to reduce HDL oxidation rate compared to lycopene rich diet [19].

HDL antioxidant properties were measured after different VOO interventions (VOO, FVOO and FVOOT) [22,23,24,25]. However, no change was found in HDL oxidation rate, HDL resistance to oxidation and glutathione peroxidase activity. To highlight, HDL oxidation rate was measured as white blood cells production of dihydrorhodamine 123 [22].

### 3.5. HDL Monolayer Fluidity

HDL monolayer fluidity was analyzed by a determination of steady-state anisotropy of 1,6-diphenyl-1,3,4-hexatriene (DHP) (Table 5). High-polyphenol content olive oil (HPCOO) [15] and extra VOO [18] consumption were found to increase HDL monolayer fluidity. However, different VOO interventions (VOO, FVOO and FVOOT) did not show effect over HDL monolayer fluidity [22,23,24,25].

Correlation models between HDL monolayer fluidity and HDL functionality-related parameters found that free HDL-C and triglyceride contents and HDL size were the main determinants of HDL monolayer fluidity [23].

### 3.6. HDL Composition

HDL lipidome is modulated by diet, even by a short-time dietary intervention. After two 4-day dietary interventions, HDL lipidome was widely modulated and fatty acids contained in HDL phospholipids were the most variable lipids by diet [17]. The 4-day MD intervention increased the quantity of phosphatidylcholine with very long chain and double bonds fatty acids, compared to baseline and to the 4-day fast food diet intervention. Fatty acid length, which was found to increase after the 4-day MD intervention, was directly related to MUFA and PUFA consumption [17]. In addition, an oleic acid rich diet, as MD, increased oleic acid and its derivatives in HDL phospholipids [19,23]. A linoleic acid rich diet increased linoleic HDL quantity, but linoleic acid binding capacity was lower than oleic acid [19].

Phenolic compounds contained in OO were found to present ability to bind to HDL particles. HDL showed higher phenolic compounds after HPCOO intervention [15] and after VOO, FVOO and FVOOT diets [23], specifically α-tocopherol, β-cryptoxanthin and coenzyme Q [23]. Lycopene rich and supplemented diets modulated HDL composition, higher HDL lycopene quantity was found after lycopene interventions [16].

HDL proteome showed to be influenced by nutritional interventions. After three interventions with VOO (VOO, FVOO and FVOOT), the HDL proteome was remodeled: 15 HDL metabolism-related proteins were identified as the most importantly modulated after the interventions, including PON3, apolipoprotein (apo)-AI, apoA-II and apoD [25]. However, no change in apo-AI quantity was found after interventions with HPCOO, LPCOO [15] and lycopene [16]. While, higher apo-AI quantity was found after a traditional MD-enriched diet enriched with nuts and VOO [14] and after three diets with VOO (VOO, FVOO and FVOOT) compared to baseline [25].

### 3.7. HDL Size

HDL size range between 8–10 nm of diameter and two groups are defined in terms of HDL size: large and small, also named as HDL3 and HDL2, respectively. The effect of different OO interventions on HDL size is shown in Table 6. Large HDL number was found to be increased after a MD enriched with VOO and with nuts [14] and after HPCOO [15] consumption. In addition, HPCOO consumption showed to decrease small HDL number. When HPCOO consumption is compared with LPCOO, large HDL number was increased and small HDL number decreased [15]. However, different VOO enriched with polyphenols (FVOO and FVOOT) showed to decrease large HDL number and to increase small HDL number [22,23,24,25]. On the other hand, extra VOO did not show capacity to modulate HDL size [18].

## 4. Discussion

HDL-C is clinically considered a CVD protective factor. However, pharmacological strategies that led to an increase in HDL-C did not reduce CVD risk [26]. The lack of effect of HDL-C raising strategies puts into question HDL-C concentrations as a protective factor against CVD. HDL-C should be in the spotlight as a CVD lowering risk factor and HDL modulations should be considered in order to predict CVD risk [3].

HDL composition is demonstrated to change in different physiological and pathological conditions [27,28]. Diseases associated to higher CVD risk, such as obesity and diabetes, remodel HDL composition and functionality. Diabetic people showed glycosylated and oxidized HDLs that presented lower HDL CEC, antioxidant and anti-inflammatory activities [29]. Moreover, lifestyle remodels HDLs and influences on their biological activities. In particular, unhealthy diets contribute to the development of dysfunctional HDLs, even in people without diseases [27]. In addition, healthy diets such as MD, have shown capacity to remodel HDL functionality even in pathological conditions, like obesity [28].

In a whole view of the data analyzed in this review, MD showed capacity to produce changes in HDL by modulating HDL functionality, oxidation, composition, and size. MD-induced changes in HDL are variable between records analyzed and not all data were comparable because the same measurements were not always performed. This variability could be influenced by population differences, healthy and high cardiovascular risk populations. Moreover, despite all records performed interventions with MD, different foods or dietary patterns were used in each record, which may contribute to different changes in HDLs, highlighting the need for greater consistency between studies in the amount of foods and nutrients administered as part of a MD. Interestingly, intervention time in the studies included in this review was found not to be a limiting factor, because even after short interventions HDL were remodeled. Several studies were conducted over different samples of the PREDIMED population, which demonstrated that HDL are widely altered by diet and that MD improves HDL quality [13,14,21].

A set of 5 studies made an intervention with VOO [13,14,18], OO or components isolated from OO, specifically polyphenols [15,22,23,24,25] and oleic acid [19]. HDL CEC was the most OO-enhanced HDL function [13,14,15,18,22,23,24,25], due, at least in part, to OO polyphenols [15,22,23,24,25]. CETP was not significantly decreased after OO interventions, with the exception of a traditional MD-enriched with VOO [14]. However, when the study population was subdivided, the new VOO group did not show changes in CETP activity [13]. LCAT activity showed significant change only after FVOOT when compared to VOO [22,23,24,25], which suggests that LCAT activity is specially modulated by polyphenols. However, a HPCOO diet did not increase LCAT activity, against baseline neither against LPCOO [15]. In addition, lycopene rich and supplemented diets were the only interventions that showed an improvement of LCAT activity compared to baseline [16]. FVOOT differs from FVOO and HPCOO in that it is enriched in thyme polyphenols. Thus, LCAT activity may be susceptible to thyme polyphenols when compared to other OO and OO polyphenols.

In regards to antioxidant functions of HDL, a traditional MD enriched with VOO and an oleic acid enriched diet lowered HDL oxidation [14,19], but no significant change in oxidation rate was found after phenol enriched VOO diets (FVOO and FVOOT). However, PON1 activity was higher after a FVOOT intervention [22,23,24,25], but no significant change was found after a traditional MD enriched with VOO [14]. FVOOT was demonstrated to increase HDL antioxidant function but not to alter HDL oxidation rate. On one hand, a traditional MD enriched with VOO decreased HDL oxidation but did not change PON1 activity. PON1 is not the only HDL antioxidant component, so the reduction on HDL oxidation rate described after a traditional MD-enriched with VOO could be due to other HDL antioxidant components, or to a MD-induced lower pro-oxidant environment, which in addition to VOO leads to lower HDL oxidation rate. On the other hand, PON1 activity could be increased by the higher presence of polyphenols on FVOOT, not altering other HDL oxidation parameters or the environment. The study population is not widely differential between interventions, since high cardiovascular risk people were included in both. However, the number of participants in the traditional MD enriched with VOO intervention was higher compared to the intervention with FVOOT, and the HDL oxidation rate was differently measured. It would be necessary to determine HDL oxidation rate after FVOO and FVOOT interventions as malondialdehyde production to be able to compare.

A HPCOO diet and an extra VOO intervention increased HDL fluidity [15,18]. There was no change in HDL monolayer fluidity in phenol enriched VOO (FVOOT and FVOO) and VOO diets [22,23,24,25]. Opposed evidences are exposed in terms of VOO and polyphenols influence on HDL monolayer fluidity. However, it is important to highlight that there were differences between the study populations. When the study population was healthy, HDL monolayer fluidity was increased by OO, but no change was found when the study population was hypercholesterolemic. OO does not benefit HDL monolayer fluidity when hypercholesterolemia is present.

Taken all data together, OO showed ability to modulate HDL functionality, and polyphenols may be very influencing components. Despite controversial results were found in HDL oxidation and monolayer fluidity, and the missing data of some enzyme activities, HDL functions could be modulated by OO. The remodeling pattern needs to be clarified, suggesting the need for further research.

EPA is an essential fatty acid, which has showed anti-inflammatory functions (complete). Fish is an EPA-rich food highly consumed in MD. An EPA enriched diet showed ability to increase HDL CEC and PON1 activity [20]. In addition, a traditional MD enriched with fish increased HDL CEC and PON1 activity and decreased CETP activity [13]. Dyslipidemia did not alter EPA-related HDL functionality improvement. Whereas, the data is not contradictory compared to interventions with VOO enriched with polyphenols (FVOO and FVOOT) in hypercholesterolemia, because PON1 activity and CEC were improved after FVOOT intervention and VOO, FVOO and FVOOT, respectively. It would be interesting to study the effect of EPA in hypercholesterolemic population over HDL oxidation rate as malondialdehyde production, to clarify if high CVD risk population is not susceptible to improve HDL oxidation rate by diet.

All records in which HDL compositional change was analyzed, HDL composition was modulated by a MD. HDL seem to be susceptible to the kind of lipid consumed. HDL lipid composition was enriched on dietary lipids. However, there is not a HDL composition change pattern by MD and no correlations could be made between HDL composition and HDL functionality.

HDL do not only vary in composition and functionality, they are subdivided by size in different types of HDLs. HDL size is variable due to HDL metabolism, since HDLs bind different components and modulate their size, as a result of HDL synthesis and catabolism, and it is also reflected in the HDL functions. HDL type in terms of size seems to influence CVD risk. Owing to the relation between HDL size and HDL functionality development, MD could change HDL size distribution. Results found by different researches are controversial, since VOO and nuts enriched diets increased large HDL quantity [14], and a HPCOO intervention increased large HDL quantity and decreased small HDL quantity, compared to baseline and to a LPCOO diet [15]. Nevertheless, two diets based on VOO enriched with polyphenols (FVOO and FVOOT) showed to have the contrary effect [22,23,24,25]. HDL size could be differently modulated when the study population is hypercholesterolemic, since hypercholesterolemia could alter OO benefits over HDL functionality.

## 5. Conclusions

In conclusion, this systematic review shows that MD influences HDL functionality, composition, and size. HDL functionality is improved by MD, in terms of enzymatic activity and CEC, also HDL antioxidant properties are improved, as HDL oxidation is reduced. There is a need to clarify MD-derived modulation of HDL size to determine HDL components which fundamentally are influenced by MD. In addition, further research is needed to determine MD specific food HDL-modulating abilities and the effect of the global MD. While, it would be interesting to clarify the MD abilities over healthy and hypercholesterolemic or dyslipidemic population separately. Taken together, MD has demonstrated to be an influencing factor over HDL quality, which indicate that MD could be a target to improve cardiovascular health via HDL modulation.

## Figures and Tables

**Table 1:** Population, intervention, comparator, outcome, and study design (PICOS) model of eligibility criteria.

Criteria	Definition
Population	Human studies: adult men and women, including healthy participants, obese and dyslipidemic patients and not including individuals with genetic diseases
Interventions	MD or MD isolated food interventions
Comparator	Comparison against baseline or comparison against different interventions
Outcomes	HDL composition changes HDL functionality changes HDL components modifications
Study design	Human pilot studies and controlled trials

HDL, high-density lipoprotein; MD, Mediterranean diet.

**Table 2:** Study characteristics.

Authors and Publication Year	Study Characteristic					Intervention
	Sample	Age	Country	Duration	Design	
A. Hernaez et al., 2019 [13]	196 individuals from the PREDIMED study	55–80	Spain	1 year	Randomized controlled trial	Traditional MD—VOO Traditional MD–nuts Traditional MD—fish Traditional MD—legumes Traditional MD—whole grains
A. Hernaez et al., 2017 [14]	296 individuals from the PREDIMED study	55–80	Spain	1 year	Randomized controlled trial	Traditional MD—nuts Traditional MD—VOO Low-fat diet
A. Hernaez et al., 2014 [15]	47 individuals from the EUROLIVE study	20–60	Germany Spain Finland	3 weeks	Randomized controlled trial	HPCOO LPCOO
N. Tanaka et al., 2014 [20]	28 dyslipidemic individuals	50–85	Japan	4 weeks	Randomized trial	Low-fat diet enriched with EPA
R. Sola et al., 2011 [21]	772 individuals from the PREDIMED study	55–80	Spain	3 months	Randomized controlled trial	Traditional MD—VOO Traditional MD—nuts Low-fat diet
J. McEneny et al., 2013 [16]	54 overweight individuals	45–55	UK	12 weeks	Randomized controlled trial	Lycopene rich diet Lycopene supplemented diet
C. Zhu et al., 2019 [17]	10 healthy individuals	18–25	California	4 days	Randomized crossover trial	Fast food diet MD
O. Helal et al., 2013 [18]	26 healthy individuals	18–75	Canada	12 weeks	Randomized trial	Extra VOO
M. Farras et al., 2015 [22] S. Fernandez-Castillejo et al., 2017 [23] M. Farras et al., 2018 [24] A. Pedret et al., 2015 [25]	33 individuals of the VOHF study	35–80	Spain	3 weeks	Randomized controlled trial	VOO FVOO FVOOT
R. Sola et al., 1997 [19]	22 healthy individuals	45–55		8 weeks	Randomized crossover trial	MD rich in oleic MD rich in linoleic

MD, Mediterranean diet; VOO, virgin olive oil; HPCOO, high-polyphenol content olive oil; LPCOO, low-polyphenol content olive oil; EPA, eicosapentaenoic acid; FVOO, functional virgin olive oil; FVOOT, functional olive oil with thyme; UK, United Kingdom.

**Table 3:** High-density lipoprotein (HDL) functionality.

Author(s) and Publication Year		Measurement			
		CEC	CETP	PON1	LCAT
A. Hernaez et al., 2019 [13]	Traditional MD—VOO	Increase	NS	NS	NM
	Traditional MD—nuts	NS	NS	Increase	
	Traditional MD—legumes	NS	Decrease	Increase	
	Traditional MD—whole-grains	Increase	NS	NS	
	Traditional—fish	Decrease	Decrease	Increase	
A. Hernaez et al., 2017 [14]	Traditional MD—VOO	Increase	Decrease	NS ^a^	NM
	Traditional MD—nuts	Increase	NS	NS	
A. Hernaez et al., 2014 [15]	LPCOO	NS	NS	NM	NS
	HPCOO	NS ^b^	NS		NS
N. Tanaka et al., 2014 [20]	Low fat diet—EPA	Increase	NM	Increase	NM
R. Sola et al., 2011 [21]		NM			
J. McEneny et al., 2013 [16]	Lycopene rich diet	NM	NS	Increase	NS
	Lycopene supplemented diet		NS	Increase	Increase
C. Zhu et al., 2019 [17]		NM			
O. Helal et al., 2013 [18]	Extra VOO	Increase	NM	NM	NM
M. Farras et al., 2015 [22] S. Fernandez-Castillejo et al., 2017 [23] M. Farras et al., 2018 [24] A. Pedret et al., 2015 [25]	VOO	NS	NM	NS	NS
	FVOO	NS		NS	NS
	FVOOT	Increase		Increase	NS ^c^
R. Sola et al., 1997 [19]	Oleic acid vs. linoleic acid rich MD	NS	NM	NM	NM

MD, Mediterranean diet; VOO, virgin olive oil; LPCOO, low-polyphenol content olive oil; HPCOO, high-polyphenol content olive oil; EPA, eicosapentaenoic acid; FVOO, functional virgin olive oil; FVOOT, functional virgin olive oil with thyme; NS, no significant change; NM, not measured. ^a^ Significant change compared to traditional MD—nuts; ^b^ significant change compared to LPCOO; ^c^ significant change compared to VOO.

**Table 4:** High-density lipoprotein (HDL) oxidation.

Author(s) and Publication Year		HDL Oxidation Rate Variation
A. Hernaez et al., 2017 [14]	Traditional MD—VOO	Decreased
	Traditional MD—nuts	NS
M. Farras et al., 2015 [22] S. Fernandez-Castillejo et al., 2017 [23] M. Farras et al., 2018 [24] A. Pedret et al., 2015 [25]	VOO	NS
	FVOO	NS
	FVOOT	NS
*R. Sola* et al., 1997 [19]	Oleic acid vs. linoleic acid	Decreased in oleic acid

MD, Mediterranean diet; VOO, virgin olive oil; FVOO, functional virgin olive oil; FVOOT, functional virgin olive oil with thyme; HPCOO, high-polyphenol content olive oil; LPCOO, low-polyphenol content olive oil; EPA, eicosapenthaenoic acid; NS, non-significant change.

**Table 5:** High-density lipoprotein (HDL) monolayer fluidity.

Author(s) and Publication Year		HDL Monolayer Fluidity Variation
A. Hernaez et al., 2014 [15]	LPCOO	NS
	HPCOO	Increased
O. Helal et al. 2013 [18]	Extra VOO	Increased
M. Farras et al., 2015 [22] S. Fernandez-Castillejo et al., 2017 [23] M. Farras et al., 2018 [24] A. Pedret et al., 2015 [25]	VOO + FVOO + FVOOT	NS
R. Sola et al., 1997 [19]	Oleic acid vs. linoleic acid	NS

LPCOO, low-polyphenol content olive oil; HPCOO, high-polyphenol content olive oil; VOO, virgin olive oil; FVOO, functional virgin olive oil; FVOOT, functional virgin olive oil with thyme; NS, no significant change.

**Table 6:** High-density lipoprotein (HDL) size variation against baseline.

Author(S) And Publication Year		HDL Size Modifications
A. Hernaez et al., 2017 [14]	Traditional MD—VOO	Increased levels of large HDLs
	Traditional MD—nuts	Increased levels of large HDLs
A. Hernaez et al., 2014 [15]	LPCOO	NS
	HPCOO ^a^	Increased levels of large HDLs Decreased levels of small HDLs
O. Helal et al. 2013 [18]	Extra VOO	NS
M. Farras et al., 2015 [22] S. Fernandez-Castillejo et al., 2017 [23] M. Farras et al., 2018 [24] A. Pedret et al., 2015 [25]	VOO	NS
	FVOO	Increased levels of small HDLs
	FVOOT ^b^	Decreased levels of large HDLs

MD, Mediterranean diet; VOO, virgin olive oil; LPCOO, low-polyphenol content olive oil; HPCOO, high-polyphenol content olive oil; FVOO, functional virgin olive oil; FVOOT, functional olive oil with thyme; HDL, high-density lipoprotein; NS, no significant change. ^a^ Significant change compared to LPCOO. ^b^ Significant change compared to VOO.

## Data Availability

Not applicable.
